# A Novel Prognostic Signature of Transcription Factors for the Prediction in Patients With GBM

**DOI:** 10.3389/fgene.2019.00906

**Published:** 2019-10-01

**Authors:** Quan Cheng, Chunhai Huang, Hui Cao, Jinhu Lin, Xuan Gong, Jian Li, Yuanbing Chen, Zhi Tian, Zhenyu Fang, Jun Huang

**Affiliations:** ^1^Department of Neurosurgery, Xiangya Hospital, Central South University, Changsha, China; ^2^Department of Neurosurgery, First Affiliated Hospital of Jishou University, Jishou, China,; ^3^Clinical Medical Research Center of Hunan Provincial Mental Behavioral Disorder, Clinical Medical School of Hunan University of Chinese Medicine, Hunan Provincial Brain Hospital, Changsha, China

**Keywords:** glioblastoma, transcription factors, prognostic signature, LHX2, MEOX2, SNAI2, ZNF22

## Abstract

**Background**: Although the diagnosis and treatment of glioblastoma (GBM) is significantly improved with recent progresses, there is still a large heterogeneity in therapeutic effects and overall survival. The aim of this study is to analyze gene expressions of transcription factors (TFs) in GBM so as to discover new tumor markers.

**Methods**: Differentially expressed TFs are identified by data mining using public databases. The GBM transcriptome profile is downloaded from The Cancer Genome Atlas (TCGA). The nonnegative matrix factorization (NMF) method is used to cluster the differentially expressed genes to discover hub genes and signal pathways. The TFs affecting the prognosis of GBM are screened by univariate and multivariate COX regression analysis, and the receiver operating characteristic (ROC) curve is determined. The GBM hazard model and nomogram map are constructed by integrating the clinical data. Finally, the TFs involving potential signaling pathways in GBM are screened by Gene Set Enrichment Analysis (GSEA), Gene Ontology (GO), and Kyoto Encyclopedia of Genes and Genomes (KEGG) enrichment analysis.

**Results**: There are 68 differentially expressed TFs in GBM, of which 43 genes are upregulated and 25 genes are downregulated. NMF clustering analysis suggested that GBM patients are divided into three groups: Clusters A, B, and C. LHX2, MEOX2, SNAI2, and ZNF22 are identified from the above differential genes by univariate/multivariate regression analysis. The risk score of those four genes are calculated based on the beta coefficient of each gene, and we found that the predictive ability of the risk score gradually increased with the prolonged predicted termination time by time-dependent ROC curve analysis. The nomogram results have showed that the integration of risk score, age, gender, chemotherapy, radiotherapy, and 1p/19q can further improve predictive ability towards the survival of GBM. The pathways in cancer, phosphoinositide 3-kinases (PI3K)–Akt signaling, Hippo signaling, and proteoglycans, are highly enriched in high-risk groups by GSEA. These genes are mainly involved in cell migration, cell adhesion, epithelial–mesenchymal transition (EMT), cell cycle, and other signaling pathways by GO and KEGG analysis.

**Conclusion**: The four-factor combined scoring model of LHX2, MEOX2, SNAI2, and ZNF22 can precisely predict the prognosis of patients with GBM.

## Introduction

Glioblastomas (GBMs) are the most common malignant tumors in the central nervous system (CNS), which accounts for 14.9% of primary CNS and 47.1% of primary brain tumors. The incidence of GBM increases with age, being most common during 75–84 years of age. It is generally associated with a poor prognosis, in which median overall survival (OS) is 15 months and 5-year survival is only about 5.5% (Ostrom et al., 2017). However, studies have shown that the prognosis varies widely among individuals. The histopathology which is commonly used in the clinic is not an ideal prognosis marker and can even lead to erroneous judgement. For the past 10 years, rapid advancement in bioinformatics has provided better tools to explore the molecular characteristics of cancer. This way, many molecular markers and molecular characterizing systems of GBM have been identified, which offers novel insights into the better understanding of progression mechanisms, diagnosis, and treatment of GBM (Lee et al., 2018). For instance, the prognostic and predictive significance of isocitrate dehydrogenase (IDH)1/2 mutation has been validated by many studies. In these studies, GBM patients with IDH1/2 mutations have notably longer OS compared with patients without (Yan et al., 2009; Beiko et al., 2014). In addition, *O* ^6^-methylguanine DNA methyltransferase (MGMT) methylation status is another important molecular marker, predicting the therapeutic effects of temozolomide (TMZ) in GBM patients (Hegi et al., 2005).

Transcription factor (TF), also known as trans-acting factor, is a protein with a unique structure that controls the rate of transcription or the production of messenger RNA (mRNA).TF can act as an activator or repressor by interacting with cis-acting elements. During eukaryotic transcription initiation, RNA polymerase II binds to TFs to form a transcription initiation complex. Transcription is a very complex process which is operated by synchronized multi-protein complexes including TFs. According to the functional characteristics of the TFs, they can be divided into two types; the first type is general TFs such as TFII family proteins which are ubiquitous and bring the RNA polymerase through binding to the promoter region near the transcription start site to turn on genes (Kadonaga, 2004). The second type is sequence-specific TFs that bind upstream of the transcription start site to promote or inhibit the expression of a particular gene. The sequence-specific TFs contain one or more DNA-binding domains and recognize specific DNA motifs near the gene to initiate their functions. TFs are involved in different biological processes such as cell proliferation, growth, differentiation, and apoptosis. Dysfunction of TFs can lead to imbalance in homeostasis, leading to a variety of diseases. Due to the complexity of transcriptional regulation, there are not many systematic studies on transcriptional regulation of GBM. This study mainly focuses on changes of transcriptome profiling in GBM, with the intention to discover key regulatory molecules which can be developed as new markers.

In this study, we have identified, established, and evaluated a scoring system with a combination of four TFs (LHX2, MEOX2, SNAI2, and ZNF22) to assess the prognosis of GBM. To achieve this, we have integrated the analysis of GBM patients’ expression profiles or sequencing data from Oncomine, Gene Expression Omnibus (GEO), TCGA, and Chinese Glioma Genome Atlas (CGGA) databases. We also provide an evidence that the expression levels of SNAI and MEOX2 are significantly associated with histopathological grade and survival time in glioma patients, indicating that these two transcriptional factors play a crucial role in the malignancy of glioma.

## Materials and Methods

### Identification of the Differentially Expressed TFs

Gene expression profile data of the SUN brain, Murat brain, GBM, and normal brain tissue in TCGA were obtained from the Oncomine (https://www.oncomine.org/resource/) database. The statistically significant differentially expressed TFs (DETFs) were identified with a fold change larger than 2. The candidate cell-specific TF markers per tissue were derived from the molecular signature database [http://software.broadinstitute.org/gsea/msigdb/gene_families.jsp, Molecular Signatures Database (MSigDB) V6.0]. The overlapped upregulated or downregulated TFs of four groups were defined as the most widely and significantly DETFs.

### Datasets

The genome-wide mRNA array expression profile of GBM patients and their corresponding clinical information, including histology, gender, age, survival information and IDH1 gene mutation status, 1p/19q codelet, GeneExp subtype, and others, were downloaded from TCGA (https://xenabrowser.net) (Goldman et al., 2019). These clinical features and mRNA expression profile of TCGA GBM array are utilized as the training dataset which includes 524 patient samples. As for the validation dataset, there are 60 samples from GSE74187, 215 samples from the CGGA GBM RNA-Seq dataset, and 157 samples from the TCGA GBM-seq dataset, which are an independent human glioma gene expression profile. The CGGA GBM RNA-Seq dataset is downloaded from the CGGA (http://cgga.org.cn/index.jsp). The GBM mRNA-seq dataset was also gained from TCGA (https://xenabrowser.net) (Goldman et al., 2019).

### Risk Model Establishment Analysis of the Detfs and Prognosis Survival of GBM

We employed the nonnegative matrix factorization (NMF) method to find the key genes and signal pathways by clustering the DETFs, which were identified in a previous step. The gene function and pathway annotation were performed using the clusterProfiler package in R (Yu et al., 2012). Univariate Cox hazard analysis was used to identify individual single genes that affect the survival of TCGA GBM patients. And then multivariate Cox regression analysis was used to establish a linear joint risk score of gene expression level (expr) using regression coefficient *β*. The risk score for each sample was calculated as follows: risk score = expr_gene1_ × *β*_gene1_ + expr_gene2_ × *β*_gene2_ + ··· + expr_gene_ *_n_* × *β*_gene_ *_n_*. The area under the receiver operating characteristic (ROC) curve (AUC) of the time-dependent risk score was calculated using the survivalROC package of R. The samples were then divided into high- and low-risk groups based on the median or the best cutoff of risk scores, for survival analysis. Next, we randomly selected half of the samples from TCGA GBM array training set to validate the efficacy of our model. After that, we conduct the external validation with the GSE74187 dataset, the CGGA dataset, and TCGA GBM RNA-Seq dataset. The correlation analysis between high- and low-risk groups towards clinical features was performed in the training set. The multivariate Cox model was constructed using the survival package for the risk score and clinical features with a *P* value < 0.05 as cutoff, and the Nomogram chart was drawn using the regplot package. The risk model was assessed by the calibration curve and AUC.

### Gene Set Enrichment Analysis

The GSEA was performed *via* the clusterProfiler package of R. The GBM samples in TCGA were divided into downregulated and upregulated groups based on the median of the risk score of the TFs. The absolute value of normalized enrichment score (NES) > 1, *P* value < 0.05, and false discovery rate (FDR) *q* value < 0.25 were defined as the statistically significant criteria. The co-expressed genes of the prognostic-related TFs identified in TCGA dataset were identified (|Spearman’s *r*| ≥ 0.4). The genes were then subjected to the clusterProfiler package for GO (biological process) and KEGG enrichment analysis, with *P* < 0.05 as the cutoff.

### Statistical Analysis

All statistical analyses were performed using SPSS 22.0 or R software. Two groups’ statistical significance was calculated using the *t*-test or non-parametric *t*-test. The chi-square test was used to analyze the correlation of the classified data. In this study, *P* < 0.05 was defined as a statistically significant cutoff. For the Cox regression analysis, the time-dependent Cox model variable test was verified using the proportional hazard hypothesis (PH hypothesis).

## Results

### Identification of the DETFs

**Figure 1:**
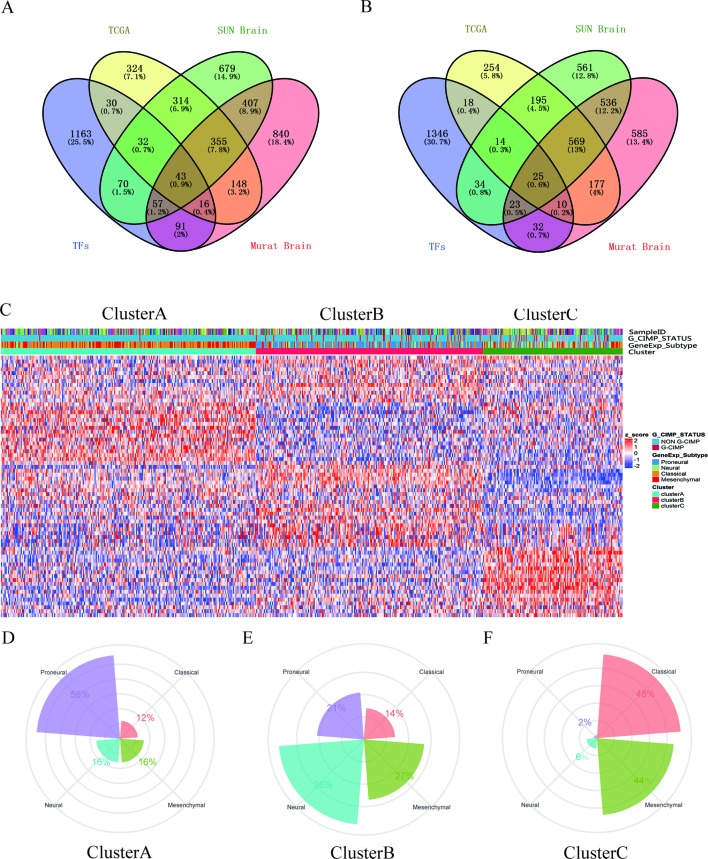
Identification of differentially expressed transcription factors (DETFs). **(A)** total of 43 significantly upregulated transcription factors were screened from the three databases of The Cancer Genome Atlas (TCGA)/SUN brain/Murat brain. **(B)** A total of 25 significantly downregulated transcription factors were screened from the three databases of TCGA/SUN brain/Murat brain. **(C)** Clusters A–C of glioblastoma (GBM) patients through 68 transcription factors using the nonnegative matrix factorization (NMF) clustering method. **(D**–**F)** Proportions of proneural, mesenchymal, classical, and neural in Clusters A–C.

**Figure 2:**
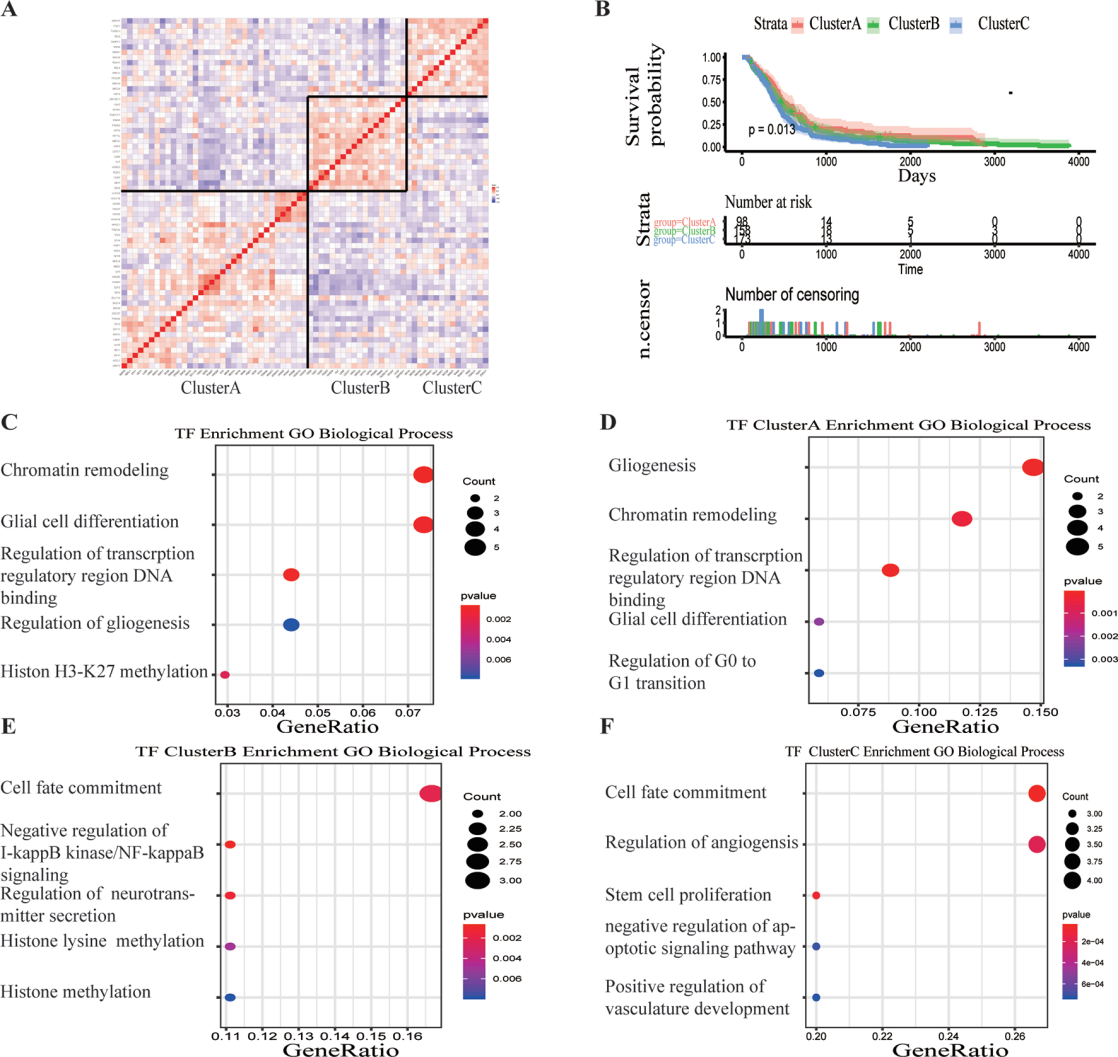
Survival analysis and gene function enrichment of Clusters A–C. **(A)** Gene expression correlation of Clusters A–C in The Cancer Genome Atlas (TCGA) glioblastoma (GBM) data. **(B)** Survival analysis of the three groups, Clusters A–C: the patients in Cluster A had the best prognosis, while those in Cluster C had the worst prognosis. **(C)** Gene Ontology (GO) (biological process) enrichment results of 68 transcription factors. **(D**–**F)** GO (biological process) enrichment results of Clusters A–C.

A total of 68 significantly DETFs were identified from TCGA/SUN brain/Murat brain database, of which 43 were upregulated and 25 were downregulated (**Table 1** and **Figures 1A, B**). Furthermore, we have obtained the gene expression profile matrix of TCGA GBM patients and have found that GBM patients can be divided into three categories using the NMF clustering method (**Figure 1C**). Representative genes of each group are shown in **Table 1**. Among them, the proneural patients in the Cluster A group were the most (56%) accounted for. Mesenchymal and classical patients were mostly in the Cluster C group, accounting for 44% and 48%, respectively. The proportions of three subtypes in the Cluster B group were very close, and the neural type accounted for a large proportion (38%) (**Figures 1D, E, F**). Correlation analysis between the 68 identified TFs has revealed that the genetic correlation among three clusters was quite good (**Figure 2A**). Patients in the Cluster A group had the best prognosis, with a median OS of 493 days. Patients in the Cluster B group had a median OS of 457 days; while patients in the Cluster C group had the worst prognosis with a median OS of 419 days (**Figure 2B**).The gene function and pathway annotation analysis by the clusterProfiler package have revealed that the most enriched pathways of the 68 TFs were chromatin remodeling, glial cell differentiation, regulation of transcription regulatory region DNA binding, and regulation of gliogenesis (**Figure 2C**). The most enriched pathways of Cluster A were gliogenesis, chromatin remodeling, regulation of transcription regulatory region DNA binding, glial cell proliferation, and regulation of G0-to-G1 transition (**Figure 2D**). The most enriched pathways of Cluster B were cell fate commitment, negative regulation of 1-kappa B kinase/nuclear factor (NF)-kappa B signaling, histone lysine methylation, and histone methylation (**Figure 2E**). The most enriched pathways of Cluster C were cell fate commitment, regulation of angiogenesis, stem cell proliferation, negative regulation of apoptotic pathway, and positive regulation of vasculature development (**Figure 2F**).

**Table 1:** Differentially expressed transcription factors (TFs).

GBM *vs* normal braint		Representative genes		
Up	Down	Cluster A	Cluster B	Cluster C
ASCL1	ARNT2	ARNT2	CBX6	HIF1A
BAZ1A	BCL11A	ASCL1	CBX7	MEF2A
CBX3	CBX6	ETV1	CHD5	MEOX2
ETV1	CBX7	HEY1	FEZF2	PDLIM5
EZH2	CHD5	LHX2	HIVEP2	PRRX1
FOXM1	FEZF2	LIMA1	HLF	RELA
HEY1	HIVEP2	RNF41	LDB2	RUNX1
HIF1A	HLF	SOX11	LDOC1	SHOX2
HMGB2	LDB2	SOX2	LMO3	SMAD1
HOXA10	LDOC1	TRIM24	MEF2C	SNAI2
HOXA5	LHX2	ZNF207	MYT1L	SNAPC1
HOXA7	LMO3	ZNF22	OPTN	TBX2
HOXB2	MED14	BAZ1A	PRDM2	TGFB1I1
HOXC10	MEF2A	BCL11A	RIMS3	TGIF1
HOXC6	MEF2C	CBX3	RUNX1T1	ZNF217
ILF3	MYT1L	EZH2	STON1	
LIMA1	NFYB	FOXM1	ULK2	
MBD2	OPTN	HMGB2	ZMYND11	
MEOX2	PRDM2	ILF3		
PDLIM5	PSIP1	MBD2		
PRRX1	RIMS3	MED14		
RARA	RNF41	NFYB		
RELA	RUNX1T1	PSIP1		
RUNX1	ULK2	RARA		
SHOX2	ZMYND11	SOX4		
SMAD1		TCF3		
SNAI2		TFAP2A		
SNAPC1		WHSC1		
SOX11		HOXA10		
SOX2		HOXA5		
SOX4		HOXA7		
STON1		HOXB2		
TBX2		HOXC10		
TCF3		HOXC6		
TFAP2A				
TGFB1I1				
TGIF1				
TRIM24				
WHSC1				
ZFAND6				
ZNF207				
ZNF217				
ZNF22				

GBM, glioblastoma.

### Construction of Prognostic Classifier From the Training Sets and Validation

**Figure 3:**
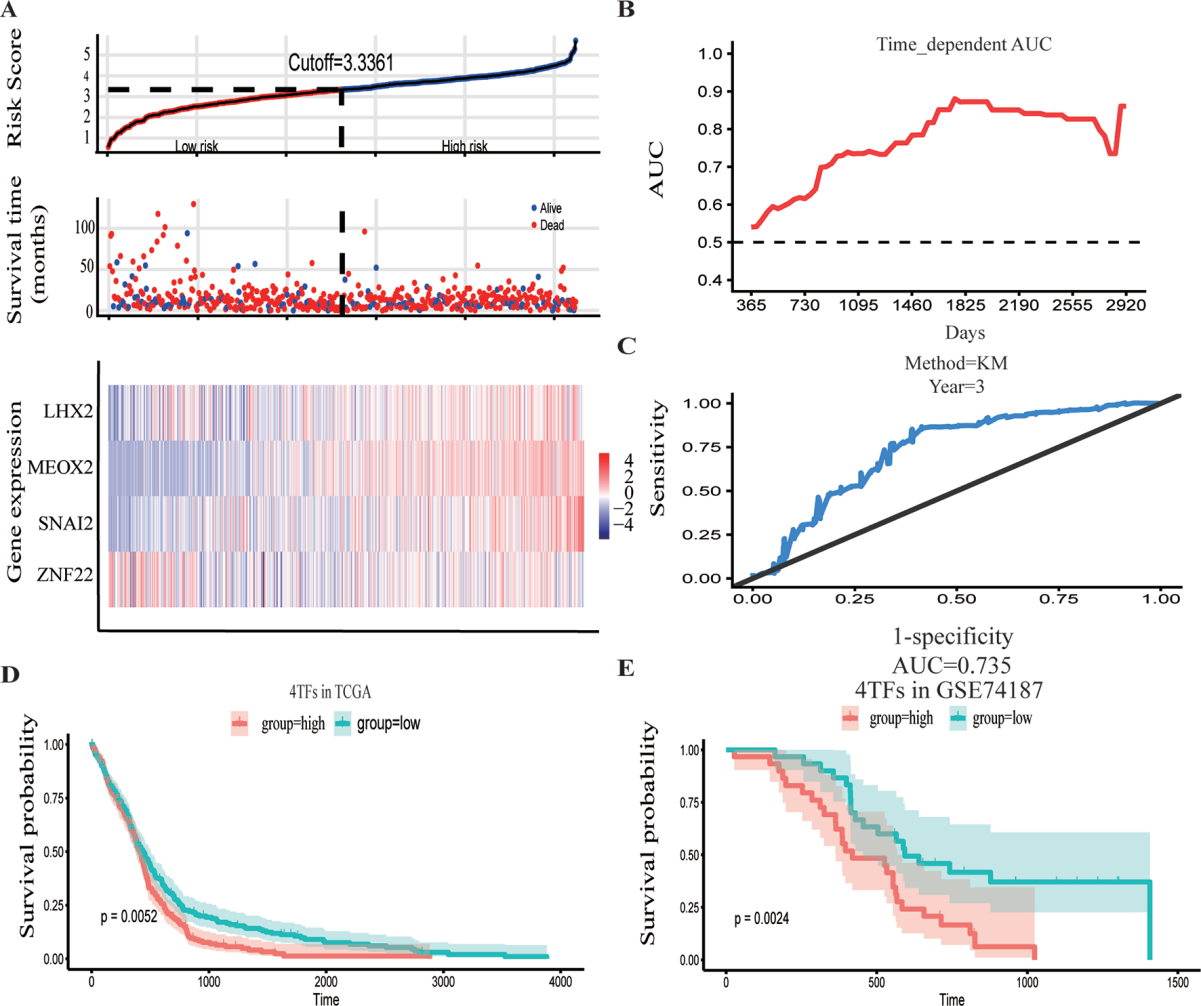
Construction and verification of the hazard assessment system. **(A)** The distribution of risk score, patient survival time and status in The Cancer Genome Atlas (TCGA) set, and heatmap of the gene risk assessment model in TCGA dataset. **(B, C)** The area under the curve (AUC) for the risk assessment model in TCGA set and time-dependent receiver operating characteristic (ROC) for predicting the 3-year survival. **(D, E)** Kaplan–Meier curves of the high-risk group and low-risk group of TCGA dataset and GSE74187 dataset.

The GBM expression profile of TCGA was used as a train dataset to screen the DETFs. Univariate Cox hazard analysis was used to identify individual single genes from 68 TFs that affect the survival of TCGA GBM patients, in which we obtained 12 statistically significant genes: ASCL1, HOXB, HOXC1, LHX2, MEOX2, RARA, RUNX1, SNAI2, SOX4, TCF3, TGIF1, and ZNF22. The 12 TFs were entered into the multivariate regression analysis. The four TFs (LHX2, MEOX2, SNAI2, and ZNF22) were inputted to the final equation, and the results indicated that these four TFs can be used as independent predictors for the prognosis of GBM. The β-cofactors of LHX2, MEOX2, SNAI2, and ZNF22 were 0.318, 0.264, 0.332, and -0.349, respectively. The joint risk score of the four TFs was calculated by substituting the coefficient into the formula. The median value was 3.3361 by ranking the risk score from low to high, which was used to divide the samples into low- and high-risk groups (**Figure 3A**). Through time-dependent ROC curve analysis, it was found that the predictive ability of the joint risk score of the four TFs for the patients’ survival prognosis gradually increased with the predicted termination time (**Figure 3B**), and the AUC of the risk score ROC curve at the predicted termination time of 3 years was 0.735 (**Figure 3C**). GBM patients were divided into high- and low-risk groups by the median value of the risk score, and the results showed that the OS time between the low- and high-risk groups was very significant (*P* = 0.0052) (**Figure 3D**). While the results of twice internal validations and the ROC curve are satisfied (**Figures S1A–D**), to validate the risk model with the external dataset, the GSE74187 dataset, the CGGA dataset, and TCGA dataset, we used the β-cooperative coefficient to calculate the joint risk score of the four TFs in each dataset that will predict the prognosis of GBM patients. With these taken together, these results manifested that the OS of GBM patients in the high- and low-risk groups was significantly different (GSE74187 *P* = 0.0024, the CGGA dataset *P* < 0.0001, and TCGA dataset *P* = 0.0055). The ROC curve also corresponds with our expectation (**Figures 3E** and **S1E–H**).

### Prognostic Value of the Integrated Classifier Is Independent of the Clinical Feature

**Figure 4:**
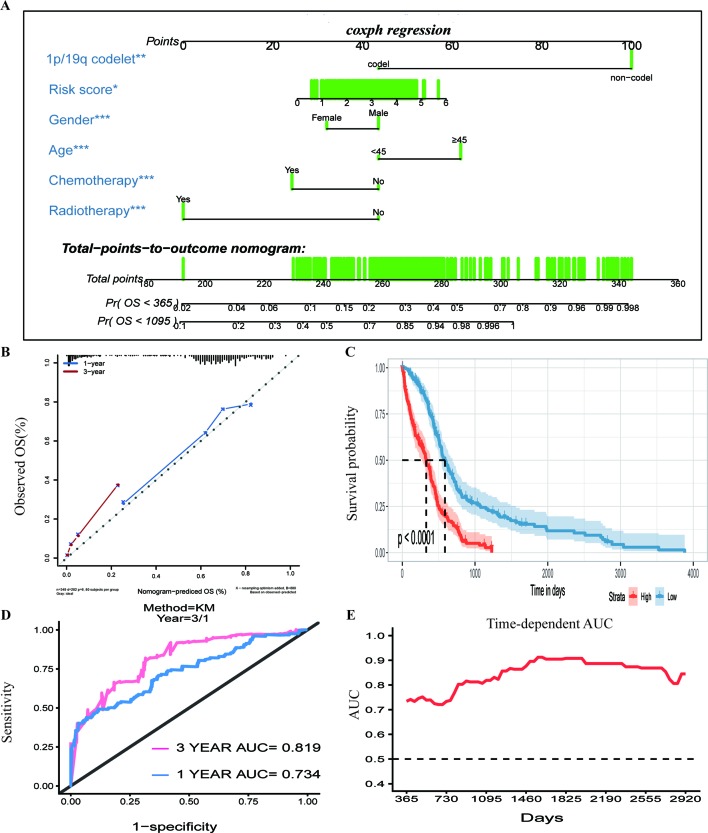
Prognostic value of the integrated classifier is independent of clinical feature. **(A)** Prognostic nomogram for glioblastoma (GBM) patients with six chief characteristics. **(B)** The calibration curve of overall survival (OS) at 1/3 year. Nomogram-predicted probability of the OS is plotted on the *x*-axis, and the observed OS is plotted on the *y*-axis. **(C)** Comparison of OS between high-risk-score group and low-risk-score group. *P < 0.05, **P < 0.01, ***P < 0.001. **(D, E)** The time-dependent receiver operating characteristic (ROC) for predicting the 1/3-year survival and area under the curve (AUC) for the risk assessment model in The Cancer Genome Atlas (TCGA) set.

To assess whether the prognostic classifier was an independent indicator in GBM patients, we analyzed the effect of each clinicopathological feature towards survival by using the Cox regression model. The multivariate regression analysis, the risk score based on TFs, age, gender, chemotherapy, radiotherapy, and 1p/19q codelet were entered into the final equation of the Cox regression model (**Table 2**). We found that the risk score based on TFs was strong and an independent predictive factor in the GBM data of TCGA (**Table 2**). Next, we constructed a nomogram that integrated TF classifiers and clinicopathological features to predict the 1-year and 3-year survival of GBM patients (**Figure 4A**). The calibration curve showed that the predicted 1-year and 3-year survival rates were closely related to the actual observed ratio (**Figure 4B**). GBM patients were divided into high- and low-risk groups by the median value of the new classifier based on the TF risk score and the clinical features. The outcome of this analysis shows that the OS of GBM patients in the high- and low-risk groups was significantly different (*P* < 0.0001) (**Figure 4C**). By calculating the AUC of the new classifier, we found that the AUC value was 0.819 at the predicted 3-year end time and 0.734 at 1-year end time (**Figure 4D**), which was higher than that using the TF classifier alone. By calculating ROC values of different times, the ROC value of the new classifier was significantly higher than that using the TF classifier only (**Figure 4E**). These results demonstrated the robust and predictive power of the new classifier based on the TF risk score and the clinical features performed better.

**Table 2:** Univariate and multivariate Cox regression analysis of factors affecting overall survival of patients in The Cancer Genome Atlas (TCGA) glioblastoma (GBM) cohort.

	Univariate analysis			Multivariate analysis		
	*P*	HR	95%CI	*P*	HR	95%CI
Risk score	<0.001	1.37	1.20–1.57	0.005	1.23	1.06–1.43
Age group (> 45)	<0.001	2.291	1.632–3.216	<0.001	2.01	1.39–2.91
Gender (Female)	0.094	0.810	0.634–1.036	0.001	0.64	0.50–0.84
Subtype						
Proneural	0.118	0.769	0.552–1.069			
Mesenchymal	0.267	1.193	0.874–1.629			
Neural	0.588	1.101	0.778–1.558			
Chemotherapy (Yes)	<0.001	0.378	0.283–0.505	<0.001	0.48	0.33–0.69
Radiotherapy (Yes)	<0.001	0.131	0.094–0.183	<0.001	0.19	0.130–0.28
IDH status (WT)	<0.001	0.321	0.196–0.524			
1p/19q codelet (non-codel)	0.046	4.24	1.026–17.52	0.005	8.54	1.89–38.5

HR, hazard ratio; IDH, isocitrate dehydrogenase; WT, wild type.

### Functional Analysis for the Prognostic Classifier of Genes

**Figure 5:**
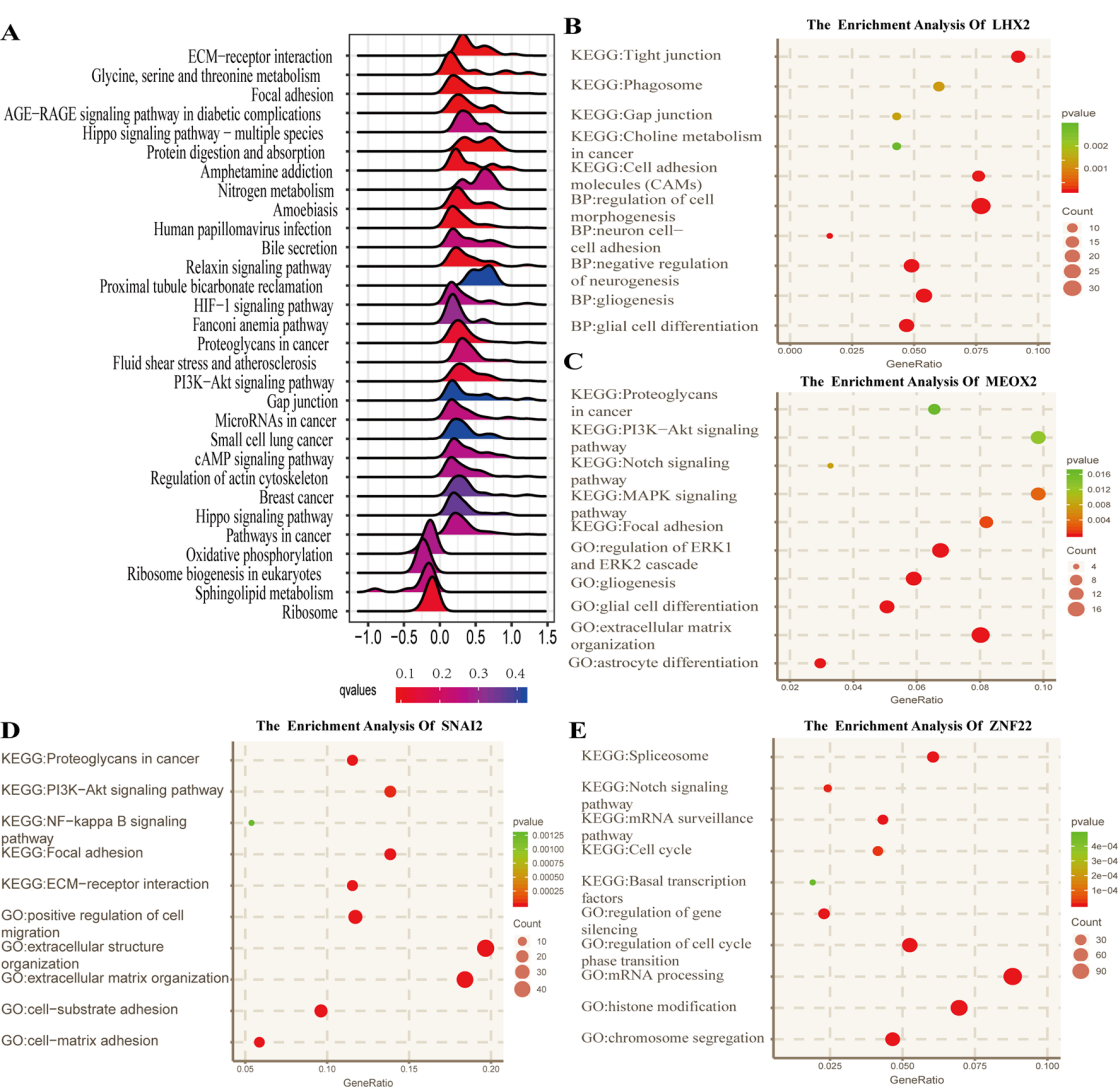
Functional analysis for the prognostic classifier of genes. **(A)** Gene Set Enrichment Analysis (GSEA) based on risk score of transcription factors is performed to identify associated pathways in Kyoto Encyclopedia of Genes and Genomes (KEGG) gene sets. **(B**–**E)** Gene Ontology (GO) (biological process) terms and KEGG pathway related to co-expressed genes of LHX2, MEOX2, SNAI2, and ZNF22 in The Cancer Genome Atlas (TCGA) dataset.

To identify the potential functional mechanisms that led to different prognosis in high- and low-risk groups, we applied functional enrichment analysis (GSEA) on identified TFs. The top 30 pathways were shown in **Figure 5A** where |NES| > 1, *P* value < 0.05, and FDR *q* value < 0.25 were used as the cutoff for identifying differentially enriched signal pathways. Pathways in cancer such as the phosphoinositide 3-kinases (PI3K)–Akt signaling pathway, hippo signaling pathway, proteoglycans in cancer, and other signaling pathways (**Figures 5B–E**) were significantly enriched in high-risk groups, which may partly explain the reason for poor prognosis in high-risk group patients. The co-expressed genes of LHX2 were mainly involved in pathways such as glial cell differentiation and cell adhesion. The co-expressed genes of MEOX2 were mainly related to the glial cell differentiation, extracellular matrix composition, cell adhesion, PI3K–Akt pathway, and other functional and pathways. The co-expressed genes of SNAI2 were mainly involved in extracellular matrix, cell invasion, cell adhesion, PI3K–Akt pathway, and NF-kappa B pathway. The co-expressed genes of SNAI2 were mainly involved in mRNA processing, histone modification, chromosome segregation, cell cycle, and Notch signaling pathway.

## Discussion

Glioma is the most common type of tumor in the brain, and its OS is still not satisfactory. In particular, the GBM patients with high-grade malignancy still have a high mortality rate (Ostrom et al., 2017). New studies are focusing on better classification, prognosis prediction, molecular mechanism, and targeted drug therapy for GBM (Touat et al., 2017). TFs play an important role in turning genes “on” and “off,” yet there are few systematic studies focusing on their roles in gliomas. By analyzing the DETFs in GBM using TCGA, SUN brain, and Murat brain datasets, we identified 68 TFs that were differentially expressed in GBM patients compared to the normal brain tissues. Using TCGA dataset as a training dataset, we found that GBM patients can be divided into three distinct subpopulations based on 68 TFs. It is well known that there is a significant heterogeneity within the malignant tumor, which leads to a large difference in its prognosis and response to various treatments. From the perspective of TFs’ expression profile, we elucidated the intrinsic differences in GBM patients, which indicated the underlining mechanisms of tumor development in different subtypes of GBM that are regulated by different signaling pathways. Our analysis showed that gliogenesis, chromatin remodeling, regulation of transcription regulatory region DNA binding, glial cell proliferation, and regulation of G0-to-G1 transition may play a major role in cancer progression in Cluster A. Cell fate commitment, negative regulation of 1-kappa B kinase/NF-kappa B signaling, histone lysine methylation, histone methylation, and so on were mainly involved in Cluster B. In the subtype of Cluster C, cell fate commitment, regulation of angiogenesis, stem cell proliferation, negative regulation of the apoptotic pathway, positive regulation of vasculature development, and so on played a more critical role. These different mechanisms of tumor progression of GBM can also explain the complex heterogeneity and differences in the prognosis. According to the subtypes of Verhaak et al. (2010), Cluster A contained more proneural subtypes, and its prognosis was better. The proportions of subtypes in Cluster B were roughly the same, while Cluster C had the most mesenchymal and classical subtypes, which may cause poor prognosis.

In order to find out which of these factors plays a key role in the prognosis of GBM, we used the Cox hazard ratio model to analyze and finally determined four independent factors (LHX2, MEOX2, SNAI2, and ZNF22) as predictors of GBM prognosis. Time-dependent ROC analysis and survival analysis found that the joint risk score based on the four TFs can accurately predict the survival prognosis. LHX2 is the major “cortical selection gene” in the cerebral cortex and plays multiple roles in different organs including the development of CNS (Chou and Tole, 2018). The relationship between LHX2 and tumor development has not yet been identified. It has been recently found that miR-124 can inhibit the migration and invasiveness of lung cancer cells by inhibiting LHX2 expression (Yang et al., 2017). Zakrzewski et al. (2015) discovered that LHX2 expressed differentially in different regions that were associated with disease progression in the underlying fibroma astrocytoma by bioinformatics, and studies have shown that this factor may play an important regulatory role in the development of tumors. The mesenchymal homeobox (MEOX) family includes two homeodomain protein+s, MEOX1 and MEOX2, with 95% sequence identity in the homologous domain, which are required for proper bone and muscle development in mouse embryos. MEOX2 is also known as a growth arrest 65-specific homeobox protein (Gax) (Northcott et al., 2017). Abnormal gene expression of MEOX2 has been found in a variety of diseases, including hepatic portal hypertension, Alzheimer disease, and cancer (Wu et al., 2005; Zeng et al., 2006). Additionally, in these diseases, MEOX2 has also been found to be associated with vascular dysfunction. MEOX2 inhibits cell proliferation and epithelial–mesenchymal transition (EMT) of vascular smooth muscle and endothelial cells (Valcourt et al., 2007). Tachon et al. (2019) demonstrated that MEOX2 expression was associated with IDH1/2 wild-type molecular subtype and was significantly correlated with the OS of all gliomas, especially in lower-grade gliomas. The Snail family of zinc finger transcriptional repressors includes three members: snai1/snail, snai2/slug, and snai3/smuc, which play key roles in EMT (Nieto, 2002; Strobl-Mazzulla and Bronner, 2012; Liu et al., 2014; Liu et al., 2017). It has been found that mRNA expression of SNAI2 was associated with histological grade and invasive phenotype in primary human glioma specimens and can be induced by epidermal growth factor receptor (EGFR) activation in human GBM cells. The overexpression of SNAI2/Slug increased the proliferation and invasion of GBM cells *in vitro* and promoted angiogenesis and tumor growth *in vivo*. Importantly, knockdown of endogenous SNAI2/Slug in GBM cells reduced invasion and increased survival in the mouse intracranial human GBM xenograft model (Yang et al., 2010). Liao et al. (2015) found that miR-203 can target SNAI2 to inhibit EMT and promote drug sensitivity and implied that targeting SNAI2 may be a potential therapeutic approach to overcome chemoresistance in GBM. In this study, we found that SNAI2 was overexpressed, and SNAI2 overexpression is characteristic for interstitial transformation, of Cluster C, proving the precision of cluster classification. ZNF22 is thought to be involved in the development of teeth (Gao et al., 2003), and its role in tumors has not been studied thoroughly. In this study, GO and KEGG analysis of DETFs revealed that these genes were mainly enriched in signal pathways such as cell migration, cell adhesion, EMT, and cell cycle, which are consistent with the studies mentioned above.

By dividing the GBM patients into high- and low-risk groups based on the four-factor joint risk score, we found that the signal pathways involved in different groups were quite different. Pathways in cancer, PI3K–Akt signaling pathway, hippo signaling pathway, and proteoglycans in cancer signaling pathways were mainly enriched in high-risk patients. These enriched malignant pathways can lead to significantly greater tumor proliferation and invasion in the high-risk group than in the low-risk group. PI3K is responsible for the conversion of PIP2 to PIP3, which activates the downstream target PKB/Akt (Chang et al., 2017; Fu et al., 2017). The PI3K pathway is usually activated by EGFR and other growth factor receptors (Zoncu et al., 2011). It was shown that the PI3K pathway was activated in almost all GBM, although only less than 15% of GBM showed activating mutations in the PI3K gene. The activation of the PI3K/Akt/mTOR pathway led to the development of GBM resistance, thereby inhibiting the therapeutic effect of chemotherapy (Li et al., 2016). The prognosis of GBM patients with activation of the PI3K–Akt pathway was terribly poor (Chakravarti et al., 2004). The hedgehog (Hh) signaling pathway, also known as hedgehog-patched (Hh-Ptch), hedgehog-Gli (Hh-Gli), or hedgehog-patched-smoothened (Hh-Ptch-Smo), is an evolutionarily conservative signaling pathway from the cell membrane to the nucleus (Skoda et al., 2018). Dysfunction or abnormal activation of the Hh signaling pathway is associated with developmental malformations and cancer, such as basal cell nevus syndrome (BCNS), sporadic basal cell carcinoma (BCC), medulloblastoma (MB), rhabdomyosarcoma, meningioma, and glioma (Taipale and Beachy, 2001; Xu et al., 2012; Skoda et al., 2018). Xu et al. (2010) found that CD44 promoted the resistance of glioma cells to reactive oxygen species-induced and cytotoxic agent-induced stress by attenuating the activation of the hippo signaling pathway. Lu et al. (2017) found that IKBKE regulated cell proliferation, invasion, and EMT of malignant glioma cells *in vitro* and *in vivo* by affecting the hippo pathway. Proteoglycans, including heparan sulfate and chondroitin sulfate proteoglycans (HSPG and CSPG, respectively), regulate the activity of many signaling pathways as well as cellular–microenvironment interactions (Nagarajan et al., 2018). Proteoglycans are the main component of the extracellular environment of the brain and regulate cell signaling and cell migration. The abnormality of proteoglycans and their modification enzymes in GBM leads to the changes of EGFR or PDGFRα signaling pathways (Wade et al., 2013). Proteoglycans are very critical for the mechanistic understanding of proteoglycan function in carcinogenic signaling and tumor microenvironment interactions in GBM and can be used to identify the new tumor biomarkers and druggable targets. These genes involved functions and pathways that are coincident with the results we found.

We analyzed the effect of each clinicopathological feature and TF risk model affecting survival by using the Cox regression model. In the multivariate regression analysis, the risk score based on TFs, age, gender, chemotherapy, radiotherapy, and 1p/19q codelet was entered into the final equation of the Cox regression model. The calibration curve of this model and AUC values indicate that the model has satisfactory accuracy. Next, we constructed a nomogram that integrated TF classifiers and clinicopathological features to predict the 1-year and 3-year survival of GBM patients. This nomogram can be used to guide doctors in judging the prognosis of GBM patients and to help them better communicate with patients.

In summary, the significantly DETFs in GBM that promote malignant progression of the tumors are mainly involved in the PI3K-Akt signaling pathway, hippo signaling pathway, proteoglycans in cancer, and other related signaling pathways. We believe that these pathways lead to poor prognosis and resistance to treatment in GBM. We have established a four-factor predictive joint risk score model that can be used to predict the prognosis of patients with GBM effectively. Based on this, two TFs closely related to the malignant progression of glioma are identified, which will provide a foundation to develop new biomarkers and targeted therapies in GBM.

## Data Availability Statement

Publicly available datasets were analyzed in this study. This data can be found here: https://xenabrowser.net. Accession: GSE74187.

## Author Contributions

QC, CH, and JH conceived and designed the idea for the manuscript and wrote the paper. HC, JLin, JLi, ZF, and XG contributed the collection, analysis, and interpretation of data. ZT and YC provided analysis tools. All authors gave approval for this version of the manuscript to be published and agree to be accountable for all aspects of the work.

## Funding

This research was supported by the National Natural Science Foundation of China (no. 81703622 and no. 81560414), China Postdoctoral Science Foundation (no. 2018M633002), Hunan Provincial Natural Science Foundation of China (no. 2018JJ3838), and Hunan Provincial Health and Health Committee Foundation of China (C2019186), and Science and Technology Department of Hunan Province (NO.2015SK2032-2).

## Conflict of Interest

The authors declare that the research was conducted in the absence of any commercial or financial relationships that could be construed as a potential conflict of interest.

## Supplementary Material

Supplementary Figure 1 The result of the internal validation dataset 1 and 2, time-dependent receiver operating characteristic (ROC) **(A**,**C)**, kaplan-Meier curves of high-risk group and low-risk group **(B**,**D)** from the TCGA GBM array dataset. As for the external validation, time-dependent receiver operating characteristic (ROC) **(E**,**G)**, kaplan-Meier curves of high-risk group and low-risk group **(F**,**H)** of the CGGA dataset and the TCGA GBM RNA-seq dataset respectively.
