# Supplementary figures and images for: A Novel Prognostic Signature of Transcription Factors for the Prediction in Patients With GBM
Source: Front Genet. 2019 Oct 1;10:906. doi: 10.3389/fgene.2019.00906 (PMC6779830; doi:10.3389/fgene.2019.00906)

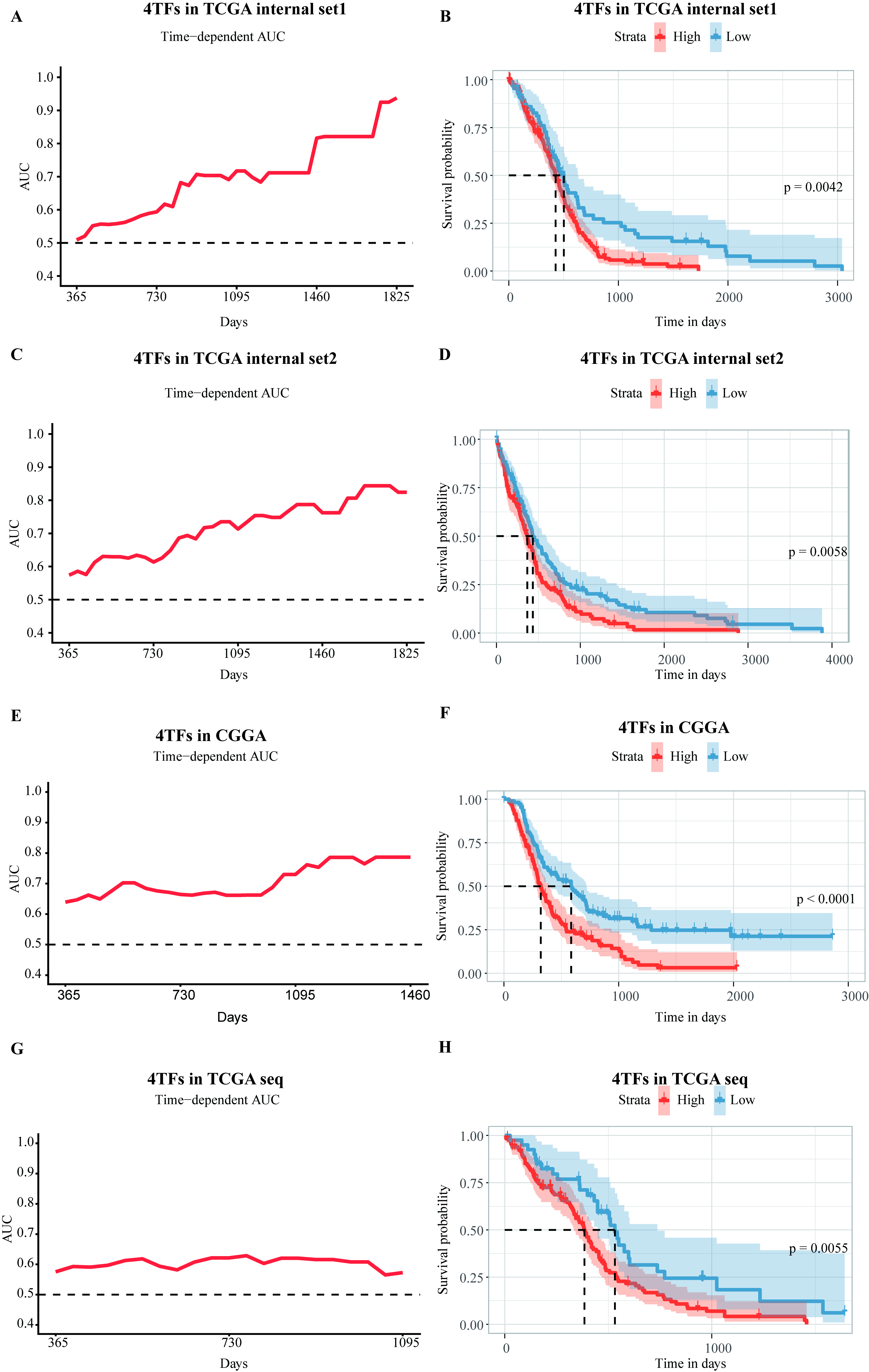

Supplement: Supplementary Figure 1 — The result of the internal validation dataset 1 and 2, time-dependent receiver operating characteristic (ROC) (A,C), kaplan-Meier curves of high-risk group and low-risk group (B,D) from the TCGA GBM array dataset. As for the external validation, time-dependent receiver operating characteristic (ROC) (E,G), kaplan-Meier curves of high-risk group and low-risk group (F,H) of the CGGA dataset and the TCGA GBM RNA-seq dataset respectively. [file Image_1.tif]
